# Impact of COVID-19 on Cervical Cancer Screening Rates Among Women Aged 21–65 Years in a Large Integrated Health Care System — Southern California, January 1–September 30, 2019, and January 1–September 30, 2020

**DOI:** 10.15585/mmwr.mm7004a1

**Published:** 2021-01-29

**Authors:** Maureen J. Miller, Lanfang Xu, Jin Qin, Erin E. Hahn, Quyen Ngo-Metzger, Brian Mittman, Devansu Tewari, Melissa Hodeib, Patricia Wride, Mona Saraiya, Chun R. Chao

**Affiliations:** ^1^Epidemic Intelligence Service, CDC; ^2^Division of Cancer Prevention and Control, National Center for Chronic Disease Prevention and Health Promotion, CDC; ^3^MedHealth Statistical Consulting Inc., Solon, Ohio; ^4^Department of Research and Evaluation, Kaiser Permanente Southern California, Pasadena, California; ^5^Department of Health Systems Science, Kaiser Permanente Bernard J. Tyson School of Medicine, Pasadena, California; ^6^Division of Gynecologic Oncology, Kaiser Permanente Orange County Women’s Health Services, Kaiser Permanente Southern California, Irvine, California; ^7^Division of Gynecologic Oncology, Riverside Medical Center, Kaiser Permanente Southern California, Riverside, California.

On March 19, 2020, the governor of California issued a statewide stay-at-home order to contain the spread of SARS-CoV-2, the virus that causes coronavirus disease 2019 (COVID-19).* The order reduced accessibility to and patient attendance at outpatient medical visits,^†^ including preventive services such as cervical cancer screening. In-person clinic visits increased when California reopened essential businesses on June 12, 2020.^§^ Electronic medical records of approximately 1.5 million women served by Kaiser Permanente Southern California (KPSC), a large integrated health care system, were examined to assess cervical cancer screening rates before, during, and after the stay-at-home order. KPSC policy is to screen women aged 21–29 years every 3 years with cervical cytology alone (Papanicolaou [Pap] test); those aged 30–65 years were screened every 5 years with human papillomavirus (HPV) testing and cytology (cotesting) through July 15, 2020, and after July 15, 2020, with HPV testing alone, consistent with the latest recommendations from U.S. Preventive Services Task Force.^¶^ Compared with the 2019 baseline, cervical cancer screening rates decreased substantially during the stay-at-home order. Among women aged 21–29 years, cervical cytology screening rates per 100 person-months declined 78%. Among women aged 30–65 years, HPV test screening rates per 100 person-months decreased 82%. After the stay-at-home order was lifted, screening rates returned to near baseline, which might have been aided by aspects of KPSC’s integrated, organized screening program (e.g., reminder systems and tracking persons lost to follow-up). As the pandemic continues, groups at higher risk for developing cervical cancers and precancers should be evaluated first. Ensuring that women receive preventive services, including cancer screening and appropriate follow-up in a safe and timely manner, remains important.

This study examined cervical cancer screening rates in women before the stay-at-home order (January 1–March 18, 2020), during the stay-at-home order (March 19–June 11, 2020), and after the stay-at-home order was lifted (June 12–September 30, 2020), compared with the same periods during January 1–September 30, 2019. Electronic medical records of women aged 21–65 years who were enrolled KPSC members for ≥1 day during this period were examined. Women with no cervix (e.g., total hysterectomy) or with a history of precancer (cervical intraepithelial neoplasia grades 2–3) or cervical cancer were excluded using relevant diagnosis and procedure codes (Supplementary Table, https://stacks.cdc.gov/view/cdc/100500). Age-specific cervical cancer screening tests per 100 person-months (cervical cancer screening rates) were calculated. Analyses were conducted using SAS (version 9.4; SAS Institute) and R (version 4.0.3; The R Foundation) software. This activity was reviewed and approved by the Kaiser Permanente Southern California Institutional Review Board, and informed consent was waived.**

The cohort included 1,455,244 women enrolled as KPSC members during January 1–September 30, 2019, and 1,492,442 women during January 1–September 30, 2020. KPSC membership enrollment was stable, with similar age group and race/ethnicity distributions in both periods (Table 1).

**TABLE 1:** Demographic characteristics of study population,* by age group^†^ and race/ethnicity — Kaiser Permanente Southern California, January 1–September 30, 2019, and January 1–September 30, 2020

Characteristic	No. (%), Jan 1–Sep 30	
	2019	2020
**Total**	1,455,244	1,492,442
**Age group, yrs**		
21–29	358,136 (24.61)	357,251 (23.94)
30–65	1,097,108 (75.39)	1,135,191 (76.06)
**Race/Ethnicity**		
Hispanic	609,057 (41.85)	617,566 (41.38)
American Indian/Alaska Native, non-Hispanic	3,032 (0.21)	3,004 (0.20)
Asian/Pacific Islander, non-Hispanic	186,841 (12.84)	186,405 (12.49)
Black, non-Hispanic	112,664 (7.74)	112,043 (7.51)
White, non-Hispanic	415,531 (28.55)	406,041 (27.21)
Multiple	7,211 (0.50)	7,304 (0.49)
Other	26,197 (1.80)	27,926 (1.87)
Unknown	94,711 (6.51)	132,153 (8.85)

* Women members of Kaiser Permanente Southern California aged 21–65 years with a cervix who do not have a history of precancer (cervical intraepithelial neoplasia grades 2–3) or cervical cancer.

^†^ Age was defined as age at mid-year (June 30). Women could be eligible in 1 or both years.

Among women aged 21–29 years, screening rates in 2020 were 8% lower before the stay-at-home order, 78% lower during the stay-at-home order, and 29% lower after the stay-at-home order was lifted compared with rates during 2019. Among women aged 30–65 years, screening rates in 2020 were 3% lower before the stay-at-home order, 82% lower during the stay-at-home order, and 24% lower after the stay-at-home order was lifted compared with rates during 2019 (Table 2). For both age groups, cervical cancer screening rates reached a nadir in April 2020 (Figure). The decreases in screening rates in 2020 compared with those in 2019 were similar across all racial and ethnic groups in KPSC.

**TABLE 2:** Comparison of cervical cancer screening rates*^,†^ before, during, and after stay-at-home order,^§^ by age group — Kaiser Permanente Southern California, January 1–September 30, 2019, and January 1–September 30, 2020

Period (relative to stay-at-home order)	Pap tests rate^†^			HPV tests rate^†^		
	Women aged 21–29 yrs			Women aged 30–65 yrs		
	2019	2020	Rate ratio^¶^ (95% CI)	2019	2020	Rate ratio^¶^ (95% CI)
Jan 1–Mar 18 (before stay-at-home order)	3.00	2.78	0.92 (0.91–0.94)	1.89	1.82	0.97 (0.95–0.98)
Mar 19–Jun 11 (during stay-at-home order)	2.63	0.59	0.22 (0.22–0.23)	1.69	0.30	0.18 (0.17–0.18)
Jun 12–Sep 30 (after stay-at-home order)	2.64	1.89	0.71 (0.70–0.73)	1.66	1.26	0.76 (0.75–0.77)

**Abbreviations**: CI = confidence interval; HPV = human papillomavirus; Pap = Papanicolaou cervical cancer test.

* Cervical cancer screening test used is a Pap test for women aged 21–29 years, and Pap test and HPV testing for women aged 30–65 years through July 15, 2020, and HPV testing alone after July 15, 2020. A combination of HPV testing and HPV and Pap (cotesting) is used in this group per U.S. Preventive Services Task Force guidelines; HPV testing rate was examined for simplicity.

^†^ Tests per 100 person-months. For women aged 21–29 years, rates were calculated as (Pap tests per person-month) x 100. For women aged 30–65 years, rates were calculated as (HPV tests per person-month) x 100.

^§^ Three contiguous but distinct periods in the year 2020 were analyzed. “Before Stay-At-Home Order” refers to all clinic encounter dates in 2020 before the state of California announced its stay-at-home executive order on March 19, 2020 (i.e., January 1–March 18, 2020). “During Stay-At-Home Order” refers to the entire period in which the state stay-at-home order was in effect, from the announcement of the order to the reopening of most essential businesses in Phase 3 of the reopening plan supervised by the California Department of Public Health (March 19–June 11, 2020). “After Stay-At-Home Order ” is inclusive of all dates after the reopening until the study cutoff date (June 12–September 30, 2020).

^¶^ 2020 Rate/2019 Rate.

**FIGURE:**
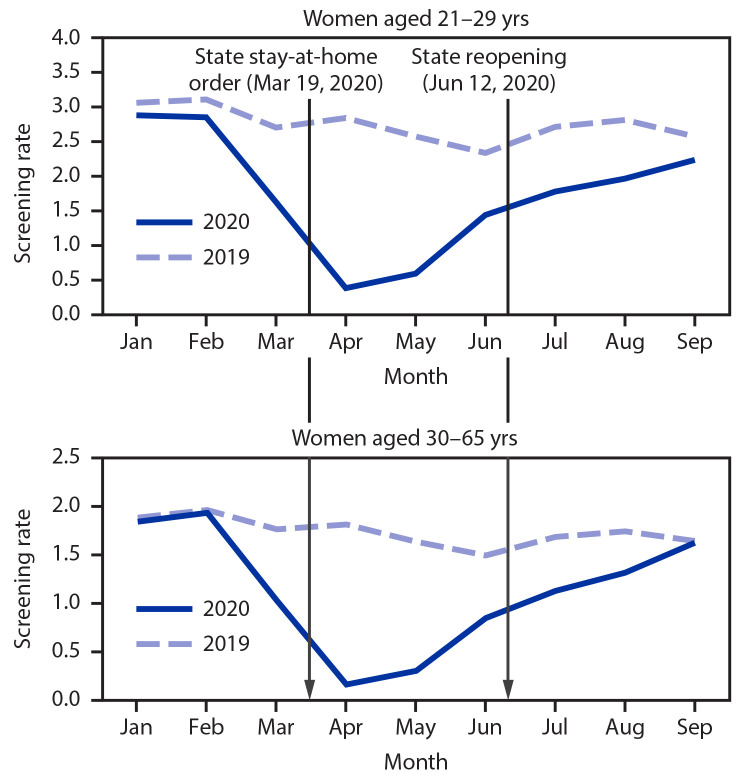
Routine cervical cancer screening rates*^,†^ among women aged 21–65 years in a large integrated health care system, by age group — Kaiser Permanente Southern California, January 1–September 30, 2019, and January 1–September 30, 2020 * Cervical cancer screening test used is Pap test for women aged 21–29 years, and Pap test and human papillomavirus (HPV) testing for women aged 30–65 years through July 15, 2020, and HPV test alone after July 15, 2020. ^†^ Tests per 100 person-months. For women aged 21–29 years, rates were calculated as (Pap tests per person-month) x 100. For women aged 30–65 years, rates were calculated as (HPV tests per person-month) x 100.

## Discussion

KPSC patient data provided an opportunity to evaluate the impact of the COVID-19 pandemic on cervical cancer screening because of the availability of a large volume of data from a diverse population and capacity of detailed monitoring and reporting. Cervical cancer screening rates at KPSC were substantially lower during the COVID-19 pandemic than during the comparable period in the preceding year. Screening rates declined in both routinely screened age groups during the stay-at-home order compared with rates during 2019, with similar declines across all racial and ethnic groups. Rates are compatible with findings of decreased cancer screening rates during 2020 in other parts of the United States (*1*–*4*). For example, the electronic health record vendor Epic Systems Corporation reviewed 2.7 million patient records from 39 organizations spanning 23 states and found a 67% decline in mean weekly cervical cancer screening volume during spring 2020, an estimated 40,000 delayed or missed screenings compared with equivalent weeks during spring 2017–2019 (*1*). One model of screening in the United Kingdom showed that a 6-month screening disruption could lead to an increased risk for cervical cancer (*5*). Such findings raise questions about how to prioritize screening of women who are overdue for screening or build screening capacity.

The COVID-19 pandemic has posed extraordinary challenges for providers and patients to maintain cancer screening (*6*). During the stay-at-home order, California cancelled elective surgeries, including some gynecologic procedures. At KPSC, although outpatient clinics never closed, and screening visits could be scheduled, in-person visits were made largely for urgent medical issues. While providing care, clinic staff members and providers faced challenges implementing COVID-19 protocols (e.g., COVID-19 prescreening, maintenance of physical distancing, use of personal protective equipment, and disinfecting surfaces and equipment).^††^ Patients experienced new barriers to access (e.g., new work and childcare schedules) and fear of SARS-CoV-2 infection from community exposure. KPSC offered telehealth appointments as an option during the stay-at-home order to maximize patient and staff member safety, resulting in a sharply increased number of telehealth visits.^§§^ Patient reluctance to come for in-person visits decreased after reopening, as providers became accustomed to new protocols and patients increased their activity outside the home. These factors likely accounted for the increase in screening rates after reopening.

The COVID-19 pandemic has highlighted a critical need for effective cancer screening methods for patients who cannot or prefer not to have in-person appointments. For colorectal cancer screening, KPSC has been using self-sampling fecal immunochemical test (FIT) kits available by mail or pharmacy and has continued mailing these to patients’ homes during the pandemic without interruptions. This approach might serve as a model for future cervical cancer screening through self-collected samples for HPV testing. The Food and Drug Administration has not yet approved self-sampling for HPV tests, but the evidence base for self-sampling demonstrates good accuracy and high acceptability among women (*7*). Self-collected HPV testing improves screening participation among women who are underscreened (*8*). Adoption of self-sampling for HPV testing might help maximize patient safety and overcome the barrier of fear of SARS-CoV-2 infection from clinic visits. However, for women who have abnormal screening results, follow-up care at a clinic could remain a challenge.

The findings in this report are subject to at least three limitations. First, it is possible that some tests considered screening tests were actually for surveillance of women with a history of cervical precancers or abnormal screening results, although women with a known history of cervical precancer and cancer were excluded. However, this potential misclassification is likely to be similar for 2019 and 2020, and thus unlikely to affect the comparisons. Second, the KPSC findings might not be generalizable to other health care settings, given differences in regional and clinic policies and individual patient health insurance status and access. KPSC is an integrated health system with an organized cervical cancer screening program through which women receive invitations to obtain screening at appropriate intervals; these continued during the stay-at-home order. Although the decreases in cervical cancer screening rates in 2020 compared with those in 2019 at KPSC were similar across all racial and ethnic groups, this might not be the case in other settings. Cervical cancer incidence and mortality rates are disproportionately higher in Hispanic women and non-Hispanic Black women than in non-Hispanic White women because of existing disparities.^¶¶^ A larger decrease and a slower return in screening rates might be experienced in other health care settings, such as safety-net clinics with persons who are medically underserved, where the level of access and health systems interventions (e.g., patient reminder systems, telemedicine) vary significantly across groups and individual persons (*9*). Finally, the screening history of women who returned for cervical cancer screening after reopening was unknown. It is unclear whether women who came for screening after the stay-at-home order was lifted in June 2020 were those who missed screening during the stay-at-home order or those who were due for screening after the reopening. Such information is needed to determine whether women who are due for cervical cancer screening are screened.

The COVID-19 pandemic is ongoing; California implemented limited and regional stay-at-home orders during November 21, 2020–January 25, 2021, affecting all California counties with widespread community transmission of SARS-CoV-2.***^,^^†††^ During the pandemic and postpandemic periods, evidence-based approaches to education, health promotion, and information dissemination could be used to convey the importance of screening for cervical cancers and precancers. Continued monitoring of women in different clinical settings is needed to address delays and interruptions to cancer screening. Health systems might triage women for return screening appointments based on risk level and screening history, including enhanced efforts to reach those who are past due for screening or who need follow-up (*10*). Focusing public health interventions on bringing higher risk populations back to screening first, such as those with abnormal results or increased risk for precancers and cancers, is suggested per guidance from the American Cancer Society, the American College of Obstetricians and Gynecologists,^§§§^ and the American Society for Colposcopy and Cervical Pathology.^¶¶¶^ As the pandemic continues, public health interventions to address decreases in cancer screening rates will be critical to avoid increased incidence of advanced cancers because of delayed detection.

**Summary**

**What is already known about this topic?**

Cancer screening rates, including cervical cancer screening rates, have declined during the COVID-19 pandemic.

**What is added by this report?**

During California’s stay-at-home order, cervical cancer screening rates among approximately 1.5 million women in the Kaiser Permanente Southern California (KPSC) network decreased approximately 80% compared with baseline. The decrease was similar across all racial/ethnic groups of KPSC and returned to near normal after reopening.

**What are the implications for public health practice?**

Sustained disruptions could lead to increased risk for cervical cancers and precancers. During a pandemic, bringing populations at higher risk back to screening first, such as those with abnormal results or increased risk for precancers and cancers, is important.

## Footnotes

*
https://covid19.ca.gov/img/N-33-20.pdf

†
https://www.cdph.ca.gov/Programs/CID/DCDC/Pages/COVID-19/ResumingCalifornia%E2%80%99sDeferredandPreventiveHealthCare.aspx

§
https://covid19.ca.gov/safer-economy

¶ https://www.uspreventiveservicestaskforce.org/uspstf/recommendation/cervical-cancer-screening. KPSC was using one of three USPSTF-recommended strategies before July 15, 2020, then switched to another HPV-based strategy after that date.

** 45 C.F.R. part 46; 21 C.F.R. part 56.

††
https://www.acs4ccc.org/wp-content/uploads/2020/10/ACS_Guidance_on_Cancer_Screening-Report_October-2020_Toolkit.pdf

§§
https://emergency.cdc.gov/coca/calls/2020/callinfo_120820.asp

¶¶
https://gis.cdc.gov/Cancer/USCS/DataViz.html

***
https://www.cdph.ca.gov/Programs/CID/DCDC/Pages/COVID-19/limited-stay-at-home-order.aspx

†††
https://covid19.ca.gov/stay-home-except-for-essential-needs

§§§
https://www.acog.org/womens-health/faqs/coronavirus-covid-19-and-womens-health-care

¶¶¶
https://www.asccp.org/covid-19
